# Immune Checkpoint Inhibitors as a Neoadjuvant/Adjuvant Treatment of Muscle-Invasive Bladder Cancer: A Systematic Review

**DOI:** 10.3390/cancers14102545

**Published:** 2022-05-21

**Authors:** Biagio Barone, Armando Calogero, Luca Scafuri, Matteo Ferro, Giuseppe Lucarelli, Erika Di Zazzo, Enrico Sicignano, Alfonso Falcone, Lorenzo Romano, Luigi De Luca, Francesco Oliva, Benito Fabio Mirto, Federico Capone, Ciro Imbimbo, Felice Crocetto

**Affiliations:** 1 Department of Neurosciences, Reproductive Sciences and Odontostomatology, University of Naples “Federico II”, 80131 Naples, Italy; biagio.barone@unina.it (B.B.); enrisici90@gmail.com (E.S.); alfonso.falcone01@gmail.com (A.F.); loryromano@hotmail.it (L.R.); luigideluca86@gmail.com (L.D.L.); fmirto22@gmail.com (B.F.M.); fedecapone@outlook.it (F.C.); ciro.imbimbo@unina.it (C.I.); 2 Department of Advanced Biomedical Sciences, Federico II University, 80131 Naples, Italy; armando.calogero2@unina.it; 3 Servicio de Cirugía General, Xerencia de Xestión Integrada de Santiago (XXIS/SERGAS), 15706 Santiago de Compostela, Spain; 4 Oncology Unit, Hospital ‘Andrea Tortora,’ ASL Salerno, 84016 Pagani, Italy; lucaluca@hotmail.it; 5 Division of Urology, European Institute of Oncology, IRCSS, Milan, Via Ripamonti 435, 20141 Milan, Italy; matteo.ferro@ieo.it; 6 Urology, Andrology and Kidney Transplantation Unit, Department of Emergency and Organ Transplantation, University of Bari, 70124 Bari, Italy; giuseppe.lucarelli@inwind.it; 7 Department of Medicine and Health Sciences “V. Tiberio”, University of Molise, 86100 Campobasso, Italy; erika.dizazzo@unimol.it; 8 Department of Urology, Policlinico di Abano, 35031 Abano Terme, Italy; francesco.oliva88@gmail.com

**Keywords:** muscle-invasive bladder cancer, adjuvant, neoadjuvant, immune checkpoint inhibitors

## Abstract

**Simple Summary:**

Bladder cancer is the ninth most common cancer worldwide. Immune checkpoint inhibitors, a novel class of immunotherapy drugs that restore natural antitumoral immune activity, have been applied to improve the overall survival and to reduce the morbidity and mortality of bladder cancer both in neoadjuvant and adjuvant settings. However, some patients do not respond to checkpoint inhibitors. Consequently, the capability for identifying patients eligible for this type of immunotherapy represent one of the efforts of ongoing studies. We aim to summarize the most recent evidence on immune checkpoint inhibitors in neoadjuvant and adjuvant setting in the treatment of muscle-invasive bladder cancer.

**Abstract:**

Bladder cancer is the ninth most common cancer worldwide. Over 75% of non-muscle invasive cancer patients require conservative local treatment, while the remaining 25% of patients undergo radical cystectomy or radiotherapy. Immune checkpoint inhibitors represent a novel class of immunotherapy drugs that restore natural antitumoral immune activity via the blockage of inhibitory receptors and ligands expressed on antigen-presenting cells, T lymphocytes and tumour cells. The use of immune checkpoint inhibitors in bladder cancer has been expanded from the neoadjuvant setting, i.e., after radical cystectomy, to the adjuvant setting, i.e., before the operative time or chemotherapy, in order to improve the overall survival and to reduce the morbidity and mortality of both the disease and its treatment. However, some patients do not respond to checkpoint inhibitors. As result, the capability for identifying patients that are eligible for this immunotherapy represent one of the efforts of ongoing studies. The aim of this systematic review is to summarize the most recent evidence regarding the use of immune checkpoint inhibitors, in a neoadjuvant and adjuvant setting, in the treatment of muscle-invasive bladder cancer.

## 1. Introduction

Bladder cancer (BC) is the ninth most common cancer worldwide, with an estimated yearly incidence of 430,000 new cases per year [1]. BC is more likely to occur in males [2], with an age-standardized incidence rate (ASR) that is three-fold higher in developed countries (11.6 ASR for Northern America and 11.4 ASR for Western Europe) compared to less developed countries (1.6 ASR for Western Africa, 2.2 ASR for Asia and 2.5 ASR for Central America) [3]. Tobacco, which is rich in carcinogens (e.g., aromatic amines), represents a major risk factor for BC, with smokers versus non-smokers showing a 2- to 5-fold higher risk for BC [4]. Conversely, occupational exposure (e.g., dye and rubber factories) accounts for a minority of BC cases (around 5%) [5,6,7]. At diagnosis, over 75% of patients shows a non-muscle invasive cancer that can be successfully managed with conservative local treatment and surveillance; the remaining 25% of patients exhibit a muscle-invasive disease, which usually requires cystectomy, radiotherapy or palliative treatment [8]. The five-year survival of treated patients decreases from 70% in patients with localized BC to 35% in patients with locally advanced disease and/or lymph node involvement, and to 5% in those with distant metastases [9]. Bacillus Calmette–Guerin (BCG) represents the first type of immunotherapy agent approved by the Food and Drug Administration (FDA) for non-muscle invasive bladder cancer (NMIBC). Despite its proven efficacy, its mechanism of action is not yet fully understood. The internalization and presentation of BCG with the subsequent release of cytokines may induce a strong immune response via the activation of CD4+ and CD8+ lymphocytes, leading to the destruction of cancer cells via direct cytoxicity or the increased secretion of compounds as TNF-α [10,11]. Overall, BCG immunotherapy enhances the local and systemic immune response by activating antigen-presenting cells (APC), upregulating cytokine production and increasing the expression of the major histocompatibility complex (MHC) class II on urothelial cells [12,13]. Activated T-cells play a pivotal role in the antitumoral immune response, but their response can be hampered by tumour cells and the tumour microenvironment. In this regard, a key role is played by immune checkpoint molecules, such as cytotoxic T-lymphocyte antigen 4 (CTLA-4), programmed cell death 1 (PD1) and PD1 ligand (PD-L1), which have served as crucial targets for the development of novel immunotherapy agents [14]. Several immune checkpoint inhibitors are currently available for clinical use, with a variety of cancer indications [15,16]. 

In this systematic review, we summarized the most recent updates on the use of immune checkpoint inhibitors in the perioperative clinical setting of muscle-invasive BC.

## 2. Methods

**Figure 1:**
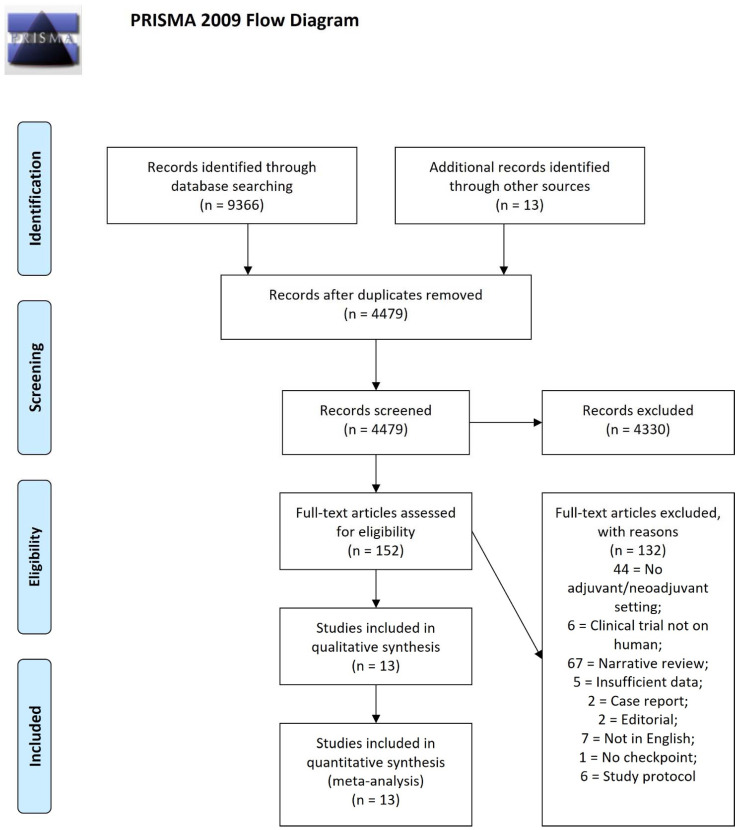
PRISMA flowchart of the reviewed manuscripts. From: Moher D, Liberati A, Tetzlaff J, Altman DG, The PRISMA Group (2009). Preferred Reporting Items for Systematic Reviews and Meta-Analyses: The PRISMA Statement. *PLoS Med* 6(7): e1000097. doi:10.1371/journal.pmed1000097 [17]. For more information, visit www.prisma-statement.org, accessed on 21 October 2021. BMJ (OPEN ACCESS) Page MJ, Moher D, Bossuyt PM, Boutron I, Hoffmann TC, Mulrow CD, et al. PRISMA 2020 explanation and elaboration: updated guidance and exemplars for reporting systematic reviews. *BMJ* 2021;372:n160. doi: 10.1136/bmj.n160 [18].

A systematic literature review was performed by querying PubMed/Medline, OVID and Scopus to identify prospective clinical trials published from January 2000 to September 2020 on immune checkpoint inhibitors therapies in muscle-invasive BC. Relevant urologic and oncologic congresses’ abstracts and journals were hand-searched to analyse further evidence. Different combinations of word algorithms were used for the literature search, which included the following entries and synonyms: Immunotherapy, PD1, PD-L1, CTLA-4, adjuvant, neoadjuvant, urothelial cancer, bladder cancer and immune checkpoint inhibitors. The search was also extended to references listed in the manuscripts included in the analysis. Data extraction was conducted to extrapolate data regarding the authors, publication year, study population, the number of participants and the treatment phase from each relevant article. The inclusion criteria were: published full articles and meta-analyses on humans; adjuvant and/or neoadjuvant therapies; patients ≥18 years of age; and English-written articles. ClinicalTrials.gov was also assessed for completed and ongoing clinical trials related to bladder cancer patients treated with immune checkpoint inhibitors. The article selection proceeded according to the Preferred Reporting Items for Systematic Reviews and Meta-Analysis (PRISMA), with the related flow diagram reported in Figure 1.

## 3. Rationale for Immune Checkpoint Inhibitors Use 

In a non-tumour-environment, immune checkpoint inhibitors normally prevent the onset of autoimmunity. During the early stages of tumour initiation, naïve T cells can migrate to the tumour microenvironment (TME) and initiate an immune response to eliminate immunogenic cancer cells [19]. In particular, T cells predominate the core of a tumour and extend beyond his invasive edge, with CD8+ T cells exerting direct cytotoxic activity and CD4+ T cells mediating antitumoral responses through the secretion of a high amount of proinflammatory cytokines (IL-2, TNF-α, INF-γ), which in turn activate macrophages and NK cells [20,21,22]. Tumour cells can evade the immune response by using two main strategies: avoiding immune recognition (via decreasing MHC-I expression and defective antigen presentation) and creating an immunosuppressive microenvironment via the production of co-inhibitory molecules [23]. Immune checkpoint molecules are expressed on immune cells that modulate the T cell response to antigens, via either the upregulation or the downregulation of immune signalling. PD-1 is a coinhibitory receptor that downregulates T cells activity and is activated by its interaction with its ligand (PD-L1), which is expressed on activated T cells, natural killer (NK) cells and APC, as well as tumour cells [24,25]. Similarly, CTLA-4 is a competitive receptor of CD28, and is upregulated on the T cell surface during the TCR/CD28/B7 interaction. The binding of CTLA-4 to B7 elicits an inhibitory signal that dampens TCR signalling, thus counteracting the stimulatory signals of CD28/B7 and TCR/MHC-II and resulting in IL-2 production suppression and T cell proliferative arrest [26]. Despite the precise mechanisms and pathways being yet to be fully elucidated, tumour cells are able to express CTLA-4 to cause the transduction of an apoptotic signal to T cells [27]. In addition, CTLA-4 tumour expression could also upregulate PD-L1 [28]. Therefore, current cancer immunotherapy strategies aim to restore T cell antitumoral activity in interacting with checkpoint molecules in order to strengthen and delimit the patient’s immune system for therapeutic purposes [29]. There are several biological factors that make immunotherapy advantageous in urothelial carcinoma, including its high mutational burden and PD-L1 expression [30]. As a result, several monoclonal antibodies blocking the ligand–receptor interactions of immune checkpoints have been tested and, in some cases, approved in urothelial cancers [31].

## 4. Neoadjuvant Setting 

The standard therapeutic approach for muscle-invasive bladder cancer consists of cisplatin-based neoadjuvant chemotherapy (NAC), followed by radical cystectomy [32,33,34]. However, up to 50% of patients are ineligible for cisplatin chemotherapy, due to multiple comorbidities, low renal function and/or previous contraindications [35,36]. In addition, NAC has been underutilized, even in eligible patients, due to possible adverse events or delays in surgery, a lack of multidisciplinary approaches or finally, the patient’s refusal [37]. The possibility for using checkpoint inhibitors in a neoadjuvant setting has clear potential advantages, due to their tolerance and efficacy, and this has been widely investigated. Multiple ongoing trials have been designed to assess the efficacy and safety of immune checkpoint inhibitors in monotherapy or in combination with other agents (such as cisplatin as well) [38].

Ipilimumab, an anti-CTLA-4 antibody that is widely used for the treatment of melanoma, was the first checkpoint inhibitor that was used in a pre-operative setting for MIBC. A study published in 2010, which enrolled cT1-T2N0M0 patients with localized urothelial carcinoma treated with two cycles of Ipilimumab (up to 10mg/kg) prior to surgery, reported encouraging preliminary results; positive urine cytology became negative, and lower-stage disease on surgical specimens compared to pre-immunotherapy transurethral resection specimens were observed. The adverse events reported were limited to rash and diarrhoea [39].

Pembrolizumab is an anti-PD-1 antibody with multiple clinical indications. In BC, it is approved as first-line treatment in cisplatin-ineligible patients with high PD-L1 expression, and in patients who received prior platinum-based treatment [40,41]. PURE-01 clinical trials included both cisplatin-eligible and -ineligible patients with cT2-3bN0M0 stage disease with high PD-L1 expression (determined using immunohistochemistry on a TURB specimen), treated with three cycles of pembrolizumab before radical cystectomy. Seventy percent of patients reported high PD-L1 expression (CPS ≥ 10%) with a median tumour mutational burden (TMB) of 11.4 mut/Mb. A complete pathologic response was associated with increasing values of CPS and TMB [42]. In particular, 42% of patients reported pT0 at surgical specimen after treatment (54.3% expressed high PD-L1 level), while 54% of patients were downgraded to non-muscle invasive tumours (65.7% expressed high PD-L1 levels) [43]. In addition, patients treated with Pembrolizumab with a higher PD-1 immune expression had 2-year progression-free survival rate of 93%, compared to 79% of patients with a lower PD-1 immune expression [44]. In addition, preliminary results reported a modest activity of Pembrolizumab in MIBC with different histologic variants, and in particular, the squamous cell carcinoma variant (SCC) and lymphoepithelioma-like variant (LEL) [45]. Although the efficacies of chemotherapy and immunotherapy have been poorly investigated so far, these data are in line with the results that are currently available on immunotherapy. Epaillard et al., reported, indeed, an overall response rate of 62.2% for chemotherapy and 22.2% for immune checkpoint inhibitor therapy (pembrolizumab in 77.8% of cases) in 46 patients with advanced or metastatic non-urothelial BC. In addition, Philip et al. referred to an overall response rate of 26% (pembrolizumab, 66% of cases; atezolizumab, 33% of cases) for metastatic non-urothelial BC, confirming the comparable efficacy of immune checkpoint inhibitors across different histological variants [46,47,48]. Controversial and lacking evidence are instead reported regarding the efficacy of chemotherapy on histologic variants, with only pure squamous cell carcinoma reporting the best outcomes compared to adenocarcinoma, small cell carcinoma and sarcomatoid variants [49]. No significant differences were reported for surgical safety, both in terms of complications and hospitalization, regardless of the robot-assisted or open radical cystectomy (RC) approach, compared to RC alone or RC after chemotherapy [50,51].

Atezolizumab is another anti-PD1/PDL1 antibody that was approved in 2016 for metastatic non-small lung cancer (NSCLC) and urothelial cancer [52]. A single-arm phase II study, the ABACUS trial, investigated the use of two cycles of atezolizumab (1200mg per cycle) every three weeks in 95 cisplatin-ineligible patients with MIBC before cystectomy. The overall pathological complete response (pCR) rate was 31% for <T3 patients, while the pCR rate was 17% in patients with T3–T4 disease. The pCR rate in patients that were positive for PD-L1 was even higher, reaching 37% despite no significant association being reported between PD-L1 expression and outcome. Pre-existing T cell immunity, and in particular, intraepithelial CD8+ T cells, was associated with a pCR rate of 40% compared to 20% for cases with an absence of intraepithelial CD8+ T cells. The one-year relapse-free survival was 79% overall, 75% in PD-L1 positive patients and 85% for patients who expressed intraepithelial CD8+ T cells. Radiological responses, according to RECIST (version 1.1) and progression before surgery occurred, respectively, in 22% and 16% of cases. Additionally, in this case, neoadjuvant treatment did not complicate surgery, with 45% of patients reporting a Clavien-Dindo of I–II, while 17% of patients reported a Clavien-Dindo of III–IV [53].

GU14-188, a phase Ib–II clinical trial, investigated the efficacy of a neoadjuvant combination of a checkpoint inhibitor (Pembrolizumab) associated with standard chemotherapy (Cisplatin/Gemcitabine) on T2-T4N0M0 BC patients. The enrolled patients were treated with 200 mg of Pembrolizumab every 3 weeks (for a total of five cycles) and 70 mg/m^2^ of cisplatin or 1000 mg/m^2^ of Gemcitabine for four cycles, followed by radical cystectomy. A robust disease downstage and control rate was reported, accounting for a pathologic non-muscle-invasive rate (PaIR) of 60%, not correlating with PD-L1 expression. Relapse-free survival, overall survival and disease-specific survival at 14 months were, respectively, 80%, 94% and 97% [54]. An interim analysis in the cisplatin-ineligible cohort reported comparable results, with a PaIR of 51.6% (57% of patients with cT2 and 47% of patients with >cT2) and a pCR of 45.2%. The estimated free survival, overall survival and disease-specific survival at 12 months were, respectively, 74.9%, 93.8% and 100%. Treatment-related adverse events included neutropenia (24%) and anaemia (13%), confirming the safety and feasibility of treatment in cisplatin-ineligible patients [55].

A Phase Ib NABUCCO clinical trial evaluated the efficacy of Ipilimumab plus Nivolumab (a human full-length immunoglobulin targeting PD1) in cisplatin-ineligible stage III urothelial cancer patients. Twenty-four patients were enrolled with a clinical T stage of T2-T4N0M0, and treated with Ipilimumab 3 mg/kg at day 1, Ipilimumab 3 mg/kg + Nivolumab 1 mg/kg at day 22 and finally Nivolumab 3 mg/kg at day 43, followed by radical cystectomy. Forty-six percent of patients reached pCR, with 58% of patients showing no residual invasive cancer (pTa or pCR) after treatment, while 8% of patients achieved a major pathological response (<10% residual vital tumour + pN0). Fifty percent of pCR was observed in patients without lymph node metastases, compared to 40% pCR in patients with clinically suspected node-positive disease. Furthermore, when patients were compared for PD-L1 expression, pCR was 73% for PD-L1 positive tumours versus 33% in PD-L1 negative tumours, with patients showing higher TMB that achieved pCR compared to non-pCR. Different from the ABACUS trial, no correlation was observed between the baseline CD8+ T cell density and the response to checkpoint inhibitors, reporting, therefore, a response to treatment independent from the presence of CD8+ T cells and inflammatory signatures. Grade 3–4 immune-related adverse events occurred in 55% of patients, which was lowered to 41% if laboratory test abnormalities were excluded as an increase of serum lipase concentration [56].

The BLASTT-1 clinical trial is currently investigating the combination of Nivolumab, Gemcitabine and Cisplatin in cT2-T4aN0-1M0 MIBC patients. The protocol included 70 mg/m^2^ of cisplatin on day 1, Gemcitabine 1000 mg/m^2^ on day 1 and day 8 and Nivolumab 360 mg on day 8, every 21 days for four cycles, followed by RC. pCR was observed in 65.8% of patients, also including N1 patients. Grade 3–4 adverse events included 20% of patients reporting neutropenia, thrombocytopenia and renal insufficiency. Follow up is currently ongoing for their progression and survival [57].

All of the reported clinical trials are currently ongoing, with the evidence described as preliminary results. In addition, several clinical trials evaluating checkpoint inhibitors or combination therapies in a neoadjuvant setting for MIBC (including radiotherapy, chemotherapy, PARP inhibition, CD73, CD137 and IDO1 targeting agents) are currently ongoing. Table 1 summarizes the main trials evaluating the neoadjuvant therapy for MIBC.

## 5. Adjuvant Setting 

Adjuvant chemotherapy after RC for pT3-4 and N+ patients without clinically detectable metastases is currently recommended in the urologic guidelines, although this is still under debate [58]. The assessed chemotherapy regimens consist of the following therapies: Cisplatin, Adriamycin and Cyclophosphamide (CAP); Cisplatin and Methotrexate (CM); Cisplatin, Methotrexate and Vinblastine (CMV); Methotrexate, Vinblastine, Adriamycin and Cisplatin (MVAC) or the substitution of Adriamycin with Epirubicin (MVEC); Gemcitabine, Cisplatin and Paclitaxel (GCP); and Gemcitabine and Cisplatin (GC) [59]. Although the advantages of adjuvant chemotherapy are mainly linked to the possibility for treating immediately possible micrometastases, the principal disadvantages of this approach are the lack of assessment of in vivo chemosensitivity, and the delay or intolerability of treatment due to postoperative morbidity. Limited evidence is indeed reported in the literature, due to limitations in the design of the studies (a small sample size or patient dropouts) [60,61]. The evaluation in this setting of checkpoint inhibitors for adjuvant immunotherapy seems to be, with their better tolerability, a reasonable alternative to standard chemotherapy regimens, with expanded indications for cisplatin-ineligible patients, or for patients with impaired renal functions. In addition, a subset of patients who received neoadjuvant chemotherapy and RC with an unfavourable prognosis (in which no standard treatments or recommendations were established) could further be addressed for adjuvant immunotherapy [62].

Currently, three randomized phase III clinical trials are ongoing. The IMvigor010 clinical trial enrolled 809 patients with high-risk disease defined as >pT2, pN+ after neoadjuvant chemotherapy or pT3 without neoadjuvant chemotherapy. Atezolizumab was administered every 3 weeks for 16 cycles or 1 year (1200 mg per cycle) in the interventional arm, evaluating disease-free survival, overall survival, biomarkers and safety. Despite an initial inconsistency in reaching its primary endpoint of disease-free survival, a recent update reported a median disease-free survival of 19.4 months for the interventional arm, compared to 16.6 months for the control arm, with a hazard ratio of 0.89. Adverse events were in line with previous studies, reporting among the most common grade 3 or 4 adverse effects, urinary tract infection, pyelonephritis and anaemia [63,64]. The CheckMate-274 clinical trial was designed to evaluate nivolumab (240 mg intravenously every 2 weeks for 1 year) compared to the placebo in high-risk MIBC after surgery, enrolling 709 patients. The primary endpoints were disease-free survival in all randomized patients, and in the subset expressing PD-L1. The median disease-free survival was 16.5 months in patients treated with nivolumab, compared to 10.8 months in patients treated with a placebo. Similarly, at 6 months, 74.9% of the nivolumab-treated and 60.3% placebo-treated patients were alive. Patients who expressed PD-L1 (>1%) reported increased recurrence-free survival, with 22.9 months in the nivolumab-treated and 13.7 in the placebo-treated, and 77% and 62.7%, respectively, being alive at 6 months. Adverse effects of grade 3 or higher were reported in 17.9% of patients treated with nivolumab, and 7.2% in the placebo group [65]. Finally, the AMBASSADOR clinical trial, which is investigating the use of pembrolizumab in MIBC after surgery, is currently ongoing, with no results being published until now. Table 2 summarizes the main trials evaluating the adjuvant therapy for MIBC. Table 3 summarizes the published clinical trials.

## 6. Predictive Biomarkers 

Some patients do not respond to checkpoint inhibitors. The possibility for identifying predictive biomarkers could increase the benefits of immunotherapy and avoid the exposure of patients to possible toxic effects where a minimal likelihood of the response is hypothesized. An ideal biomarker would be reliably reproducible, cost-effective and observer-independent, strongly correlating with clinical outcomes. Overall, different emerging biomarkers have been included in recent clinical trials: PD-L1 expression, TMB, immune gene expression profiling and tumour-infiltrating lymphocytes.

PD-L1 expression has been rigorously examined as being a potential biomarker, although the data remain inconclusive. The lack of standardization across immunohistochemical assays, plus the dynamic nature of PD-L1 expression is one of the explanations for the difficulty in the interpretation of results. The most commonly used method of PD-L1 expression assessment is currently the DAKO 22C3 assay, an immunohistochemical assay that is performed with a murine monoclonal anti-human PD-L1 antibody [66]. This assay permitted the inclusion of a standardized protocol in the determination of PD-L1 expression, measured as the percentage of tumour cells and infiltrating immune cells expressing PD-L1, and has shown, despite all previously reported limitations, good premises as a predictive biomarker. In particular, preliminary data in recent clinical trials, such as those reported in this review, suggest that patients with high PD-L1 expression have higher pCR compared to patients with low or negative PD-L1 expression that also show responses to treatment [43,53,54,56]. On this basis, PD-L1 expression, both for cancer and for immune cells, has been suggested as being a first potential predictive biomarker for testing the efficacy and sensitivity of immune checkpoint inhibitors [67]. Forty-four distinct trials involving 6664 patients with solid tumours showed, indeed, a favourable predictive response of 2.26-fold higher in patients expressing PD-L1 on the cancer cell surface, compared to PD-L1-negative patients [68]. Regarding these outcomes, three workshops were recently held in order to develop recommendations for best-practice approaches toward PD-L1 testing in urothelial cancers, in order to overcome the expression level limitations and unstandardized scoring algorithms [69].

TMB has recently been investigated as a potential biomarker to evaluate the immunotherapy response. The presence of somatic or germline mutations increases tumour-associated antigens, and consequently, tumour immunogenicity. TMB seems to be correlated to treatment responses with checkpoint inhibitors in different cancers, although the data regarding MIBC are not fully elucidated [70]. In clinical trials such as ABACUS, no correlation is reported between TMB and the response to treatment [53], while differently, in PURE-01, TMB is positively correlated with a response to treatment [43]. Therefore, TMB could predict the response to treatment, especially in addition to PD-L1 expression. However, further evidence and larger studies are required. Recently, TMB was evaluated as being a predictive biomarker in immune checkpoint inhibitor responses across multiple cancer types in over than 1600 patients, reporting high responses and long survival rates in patients who reported higher TMB. Nevertheless, the optimal predictive cut-off widely varied among different histological types, ranging from 10% to 50% [71]. Specifically, urothelial carcinomas with high TMB showed a better prognosis and a high prevalence of mutations in *TP53, PIK3CA* and *FAT4*, which could be used in combined treatments [72]. Interestingly, Tang et al., reported a radiomic-based TMB predictive model that aimed to build a pre-testing nomogram calculating the possibility of high TMB in BC patients [73].

Several studies have demonstrated a possible predictive role of the gene expression signature as immunosuppressive genes, monocyte and macrophage chemotactic genes, mesenchymal transition genes and interferon-gamma signalling. However, larger trials are required to validate those hypotheses [74,75].

In addition, as previously reported, a tumour microenvironment infiltrated by T cells could be a potential biomarker for clinical benefits from immunotherapy. The presence of CD8+ T cell infiltration has been shown to correlate with improved clinical outcomes in MIBC, due to the enhancing action of immune checkpoint inhibitors on T cells infiltrating the tumours [21]. CD4+ T cells are also included as potential predictive biomarkers; in particular, the increased expression of ICOS in anti-CTLA-4 clinical trials is possibly associated with better clinical outcomes [39].

Lastly, the use of circulating tumour DNA (ctDNA) has recently emerged as a biomarker of multiple solid tumour types. ctDNA could be obtained from blood, avoiding the shortcomings of tissue-based biopsy, in order to identify patients with molecular residual disease after surgery [76,77,78]. In addition to its role in BC surveillance, ctDNA has been also used as a marker of therapy response. Kuziora et al., by analysing ctDNA in 29 patients undergoing a 6-week treatment with durvalumab, reported a significant reduction of ctDNA (up to −2.4%) in responders compared to non-responders [79]. Similarly, Vandekerkhove et al. reported on 104 patients with metastatic BC, a more aggressive form of disease in those showing higher ctDNA levels. Moreover, Raja et al., reported an inverse correlation between ctDNA expression and overall survival in 29 patients treated with durvalumab [80,81]. Regarding a potential cut-off predicting a worse prognosis in BC patients, it has been reported, for ctDNA above 2% of the total cell-free circulating DNA, an increased rate of metastasis (>80% of cases) or of locally advanced disease (15% of cases) [82]. As reported by Powles et al., from the evaluation of ctDNA in the IMvigor010 trial, at the start of therapy, 37% of patients were positive for ctDNA and were therefore at a higher risk of disease recurrence compared to those with a ctDNA-negative status. Interestingly, patients that were positive for ctDNA had improved rates of disease-free survival and overall survival with adjuvant atezolizumab, compared to patients treated with a placebo, while no difference was reported between disease-free survival and overall survival between the treatment arms for ctDNA-negative patients. Finally, ctDNA clearance at week 6 was higher in the atezolizumab arm (18%) compared to the observation arm (4%) [83]. However, further investigations on the role of pre- and post-treatment ctDNA levels as a predictive biomarker of prognosis and overall survival in BC patients are required [84]. 

## 7. Conclusions

The advent of novel, well-tolerated immunotherapy agents that are designed to block PD-1/PD-L1 or CTLA-4 has revolutionized the perioperative treatment of MIBC. In particular, the combination of neoadjuvant immunotherapy with cisplatin-based chemotherapy has yielded complete response rates that have by far surpassed expectations, especially in selected patients (e.g., those expressing PD-L1). Future studies are warranted to define the role of immunotherapy in the perioperative setting. Predictive biomarkers that can be included in the therapeutic algorithm currently represent a highly unmet need in this scenario.

## Figures and Tables

**Table 1:** Summary of ongoing clinical trials in neoadjuvant setting.

NCT Number	Other Names	Drug	Phase	Population	Dates
**NCT04506554**	GU-176	Nivolumab + AMVAC	II	71	Study Start: August 2020 Study Completion: August 2023
**NCT04383743**	RG1006206	Pembrolizumab ± Cisplatin ± Doxorubicin ± Methotrexate ± Pegfilgrastim	II	17	Study Start: September 2020 Study Completion: February 2023
**NCT04289779**	ABATE	Atezolizumab + Cabozantinib	II	42	Study Start: May 2020 Study Completion: March 2023
**NCT04099589**	NCC2121	Toripalimab	II	64	Study Start: October 2019 Study Completion: October 2022
**NCT03978624**	LCCC1827	Pembrolizumab ± Entinostat	II	20	Study Start: March 2020 Study Completion: November 2022
**NCT03912818**	IRB-48062	Durvalumab ± Carboplatin ± Cisplatin ± Doxorubicin ± Gemcitabine ± Methotrexate ± Vinblastine	II	24	Study Start: April 2019 Study Completion: August 2022
**NCT03832673**	PECULIAR	Pembrolizumab + Epacadostat	II	38	Study Start: April 2019 Study Completion: April 2020
**NCT03773666**	BLASTT-2	Durvalumab ± Oleclumab	I	24	Study Start: February 2019 Study Completion: July 31 2022
**NCT03674424**	AURA	Avelumab	II	166	Study Start: June 2018 Study Completion: December 2022
**NCT03577132**	SeoulNUHUro_Ate	Atezolizumab	I	20	Study Start: August 2018 Study Completion: May 2022
**NCT03558087**	HCRN GU16-257	Nivolumab + Gemcitabine + Cisplatin	II	76	Study Start: July 2018 Study Completion: August 2023
**NCT03549715**	NEMIO	Durvalumab + Tremelimumab + Methotrexate + Vinblastine + Adryamicin + Cisplatin	II	120	Study Start: December 2018 Study Completion: September 2025
**NCT03534492**	NEODURVARIB	Durvalumab + Olaparib	II	29	Study Start: November 2018 Study Completion: March 16, 2020
**NCT03532451**	PrE0807	Nivolumab ± Lirilumab	I	43	Study Start: March 2019 Study Completion: September 2022
**NCT03529890**	RACE IT	Nivolumab	II	33	Study Start: February 2019 Study Completion: August 2022
**NCT03520491**	18-042	Nivolumab ± Ipilimumab	II	45	Study Start: April 2018 Study Completion: January 2021
**NCT03518320**	TAR-200-104	Nivolumab + Gemcitabine	I	13	Study Start: January 2019 Study Completion: December 2019
**NCT03498196**	H-41207	Avelumab	II	1	Study Start: December 2018 Study Completion: December 2019
**NCT03472274**	DUTRENEO	Durvalumab + Tremelimumab	II	99	Study Start: October 2018 Study Completion: December 2022
**NCT03319745**	P30CA016672	Pembrolizumab	II	20	Study Start: January 2018 Study Completion: November 2020
**NCT03234153**	NITIMIB	Durvalumab + Tremelimumab	II	6	Study Start: July 2018 Study Completion: May 2020
**NCT03212651**	PANDORE	Pembrolizumab	II	41	Study Start: October 2017 Study Completion: October 2019
**NCT02989584**	16-1428	Atezolizumab + Gemcitabine + Cisplatin	II	54	Study Start: December 2016 Study Completion: December 2021
**NCT02845323**	J1682	Nivolumab ± Urelumab	II	44	Study Start: May 2017 Study Completion: January 2021
**NCT02812420**	P30CA016672	Durvalumab + Tremelimumab	I	54	Study Start: March 2017 Study Completion: March 2022
**NCT02690558**	LCCC 1520	Pembrolizumab + Gemcitabine + Cisplatin	II	19	Study Start: May 2016 Study Completion: September 2025
**NCT02451423**	14524	Atezolizumab	II	42	Study Start: June 2016 Study Completion: December 2021
**NCT02891161**	DUART	Durvalumab	II	26	Study Start: November 2016 Study Completion: November 2021
**NCT03406650**	SAKK 06/17	Durvalumab	II	61	Study Start: May 2018 Study Completion: April 2026
**NCT03732677**	NIAGARA	Durvalumab ± Gemcitabine ± Cisplatin	III	1050	Study Start: November 2018 Study Completion: December 2025
**NCT03661320**	CA017-078	Nivolumab ± BMS-986205 ± Gemcitabine ± Cisplatin	III	1200	Study Start: October 2018 Study Completion: December 2026
**NCT03924856**	KEYNOTE-866	Pembrolizumab ± Gemcitabine + Cisplatin	III	790	Study Start: June 2019 Study Completion: January 2025
**NCT03924895**	KEYNOTE-905	Pembrolizumab ± Enfortumab Vedotin	III	836	Study Start: July 2019 Study Completion: February 2026

**Table 2:** Summary of ongoing clinical trials in adjuvant setting.

NCT Number	Other Names	Drug	Phase	Population	Dates
**NCT04138628**	TOMBOLA	Atezolizumab	II	262	Study Start: March 2020 Study Completion: November 2024
**NCT03768570**	BL13	Durvalumab	II	238	Study Start: December 2018 Study Completion: December 2024
**NCT03620435**	ML-39576	Atezolizumab	II	25	Study Start: May 2018 Study Completion: December 2020
**NCT03359239**	GCO 16-1387	Atezolizumab + PGV001 + Poly ICLC	I	15	Study Start: May 2019 Study Completion: January 2022
**NCT03244384**	AMBASSADOR	Pembrolizumab	III	739	Study Start: September 2017 Study Completion: June 2025
**NCT03171025**	NEXT	Nivolumab	II	28	Study Start: July 2017 Study Completion: June 2024
**NCT02897765**	NT-001	NEO- PV-01 + Nivolumab	I	55	Study Start: October 2016 Study Completion: May 2020
**NCT02450331**	IMvigor010	Atezolizumab	III	809	Study Start: October 2015 Study Completion: May 2022
**NCT02632409**	CheckMate 274	Nivolumab	III	700	Study Start: February 2016 Study Completion: November 2026

**Table 3:** Summary of published clinical trials.

NCT Number	Title	Other Names	Drug	Phase	Population	Dates
**NCT03387761**	Neo-Adjuvant Bladder Urothelial Carcinoma Combination-immunotherapy	NABUCCO	Ipilimumab + Nivolumab	I	54	Study Start: January 2018 Study Completion: June 2021
**NCT02662309**	Preoperative MPDL3280A in Transitional Cell Carcinoma of the Bladder	ABACUS	Atezolizumab	II	96	Study Start: February 2016 Study Completion: July 2020
**NCT02736266**	Neoadjuvant Pembrolizumab for Muscle-invasive Urothelial Bladder Carcinoma	PURE-01	Pembrolizumab	II	90	Study Start: February 2017 Study Completion: December 2019
**NCT02365766**	Neoadjuvant Pembrolizumab in Combination With Gemcitabine Therapy in Cis-eligible/Ineligible UC Subjects	GU14-188	Pembrolizumab ± Gemcitabine ± Cisplatin	I	83	Study Start: May 2015 Study Completion: July 2021
**NCT03294304**	BLASST-1 (Bladder Cancer Signal Seeking Trial): Nivolumab, Gemcitabine and Cisplatin in Treatment of Muscle Invasive Bladder Cancer (MIBC) Undergoing Cystectomy	BLASST-1	Nivolumab + Gemcitabine + Cisplatin	II	43	Study Start: January 2018 Study Completion: December 2020
